# Postoperative weightbearing timing after distal femur osteotomy is not associated with rates of union or complications: A systematic review and meta‐analysis

**DOI:** 10.1002/ksa.70340

**Published:** 2026-02-16

**Authors:** Nicholas A. Apseloff, Tarik Taoufik, Christopher C. Kaeding, Robert A. Magnussen, David C. Flanigan, Jelle P. van der List

**Affiliations:** ^1^ Department of Orthopaedic Surgery and Sports Medicine The Ohio State University Wexner Medical Center Columbus Ohio USA; ^2^ Sports Medicine Research Institute The Ohio State University Wexner Medical Center Columbus Ohio USA; ^3^ The Ohio State University College of Medicine Columbus Ohio USA

**Keywords:** distal femoral osteotomy, lateral knee osteoarthritis, osteotomy, weightbearing

## Abstract

**Purpose:**

To assess the outcomes and complication rates of early weightbearing (either immediate weightbearing as tolerated or partial weightbearing) and delayed weightbearing (an initial period of non‐weightbearing or toe‐touch weightbearing) following distal femoral osteotomy (DFO).

**Methods:**

A systematic literature search using PubMed, Embase and Cochrane Reviews was performed. Inclusion criteria were studies reporting on outcomes and complications after DFO with a minimum 1‐year follow‐up. Methodologic quality of studies was assessed using the methodological index for non‐randomised studies (MINORS) criteria. Data collection included incidence of nonunion, delayed union, loss of fixation or deformity correction, knee stiffness, venous thromboembolism (VTE) and patient‐reported outcome measures (PROMs). Meta‐analysis was performed utilising random effects models, with statistically significant results denoted by a *p*‐value < 0.05.

**Results:**

Twenty‐six studies (23 level IV and 3 level III) with 814 patients were included (mean age 42 years, mean follow‐up 5.2 years). All but one study (25/26 [96.2%]) had moderate quality methodology. Statistical comparison was limited by low event frequency, and thus no statistically significant associations were identified, and *p*‐values were omitted. The overall complication rates were nonunion 2.5% (95% confidence interval [CI] 1.6%–3.8%), delayed union 0.6% (95% CI 0.1%–3.3%), loss of fixation or deformity correction 1.4% (95% CI 0.5%–3.5%), knee stiffness 2.9% (95% CI 1.4%–6.1%), VTE 0.9% (95% CI 0.3%–2.3%). Validated PROMs were reported in 11 of 26 studies (42%) using heterogeneous instruments, precluding quantitative pooling and meta‐analysis.

**Conclusions:**

There were relatively low overall mean rates of delayed union, nonunion, loss of fixation or deformity correction, and VTE after DFO, regardless of an early or delayed post‐operative weightbearing protocol. Due to limited comparative data and the risk of selection bias, definitive conclusions cannot be drawn regarding the safety of early weightbearing after DFO, underscoring the need for prospective controlled studies.

**Level of Evidence:**

Level IV.

**Abbreviations**
ACL: anterior cruciate ligament
CI: confidence interval
DFO: distal femoral osteotomy
DVT: deep vein thrombosis
GRADE: Grading of Recommendations, Assessment, Development and Evaluation
LOW: lateral opening wedge
MCW: medial closing wedge
MINORS: Methodological Index for Non‐Randomised Studies
PRISMA: Preferred Reporting Items for Systematic Reviews and Meta‐Analyses
PROMs: patient‐reported outcome measures
VTE: venous thromboembolism

## INTRODUCTION

Distal femoral osteotomy (DFO) is a surgical procedure used to correct coronal plane deformities of the knee joint [27]. DFOs are most commonly indicated for patients with unicompartmental knee osteoarthritis, post‐traumatic knee malalignment, or to offload a compartment of the knee undergoing a concomitant procedure (e.g., cartilage restoration or meniscus allograft transplant) [16, 30]. Despite DFO being a well‐established surgical procedure, postoperative rehabilitation and timing of weightbearing after surgery are still debated. A 2024 European expert consensus recommended ‘extra caution’ for early weightbearing after DFO, but no consensus was achieved for a timeframe to full weightbearing [35].

Traditionally, delayed weightbearing protocols after DFO have been utilised to minimise the risk of complications such as loss of deformity correction, delayed union and nonunion [2, 7, 8, 9, 12, 17, 20, 22, 33, 36, 38, 39, 44, 46, 49]. However, prolonged nonweightbearing protocols may theoretically contribute to knee stiffness, muscle atrophy, delayed return to function and venous thromboembolism (VTE). With modern fixed‐angle rigid locked plating techniques, earlier weightbearing after DFO may be safe [10, 14, 15, 18, 25, 26, 29, 31, 32, 34, 43]. Current decisions on postoperative weightbearing after DFO are often based on surgeon preference or institutional protocols rather than high‐level evidence.

The primary aim of this systematic review was to compare the intervention of early weightbearing (immediate weightbearing as tolerated [WBAT] or partial weightbearing [PWB]) versus delayed weightbearing (an initial period of non‐weightbearing [NWB] or toe‐touch weightbearing [TTWB]) on the outcome of nonunion rate after DFO. Secondary aims were to compare early weightbearing versus delayed weightbearing on the outcomes of delayed union, loss of fixation or deformity correction, knee stiffness, VTE and patient‐reported outcome measures (PROMs) after DFO. It was hypothesised that there would be no difference in rates of nonunion, delayed union, loss of fixation or deformity correction, knee stiffness, VTE and PROMs between early and delayed weightbearing after DFO.

## METHODS

The Preferred Reporting Items for Systematic Reviews and Meta‐Analyses (PRISMA) guidelines were followed for this systematic review, and the study was registered in PROSPERO (CRD420251031147). There was no funding or conflict of interest for this study.

### Literature search

The electronic databases of PubMed, Embase and Cochrane were searched on 24 March 2025 for studies reporting on outcomes following DFO with different postoperative weightbearing regimens using the search algorithm ‘(distal femoral osteotomy OR distal femur osteotomy OR DFO OR (valgus osteotomy AND knee)) AND ((early OR delayed OR full OR accelerated OR non OR permissive) AND (weightbearing OR weight bearing OR weight‐bearing OR rehabilitation))’. The search was updated on 24 January 2026, to ensure inclusion of all relevant studies published during the interim. After removal of duplicates, two reviewers independently reviewed the title and abstract for potential inclusion, and the full texts of the potential studies were reviewed for final inclusion. References of included studies were screened, and five additional studies were identified for inclusion [1, 5, 13, 28, 48]. If disagreement occurred, a third reviewer was consulted.

Inclusion criteria consisted of studies (I) reporting on DFO for valgus knee alignment; (II) using plate internal fixation; (III) for the indication of coronal malalignment or osteoarthritis; (IV) adult patients (age ≥ 18 years); (V) reporting outcomes that included incidence of delayed union (absence of radiographic bony union by 6 months postoperatively) or nonunion (absence of radiographic bony union by 12 months postoperatively) [3, 23], loss of fixation or deformity correction, knee stiffness (defined by a post‐operative decrease in range of motion compared to pre‐operatively [46], or return to surgery for manipulation under anaesthesia and/or lysis of adhesions [11]), VTE and PROMs; (VI) with minimum four cases; (VII) with minimum 1 year postoperative follow‐up; and (VIII) English, French, or German language. Exclusion criteria consisted of (I) osteotomy fixation by percutaneous means, external fixator, or only cast immobilisation; (II) patients with congenital limb deformity conditions (e.g., cerebral palsy, arthrogryposis); (III) reviews, biomechanical studies, meta‐analyses, case reports; (IV) primary indication of derotational osteotomy; (V) double level osteotomy (combined DFO + high tibial osteotomy).

### Quality assessment

For all studies, the level of evidence was categorised using the adjusted Oxford Centre for Evidence‐Based Medicine grading system [47]. For all studies, methodological study quality was assessed using the methodological index for non‐randomised studies (MINORS) criteria, for which the first 8 criteria were used for non‐comparative studies and all 12 criteria for comparative studies [41]. The Grading of Recommendations, Assessment, Development and Evaluation (GRADE) approach was used to grade quality of evidence and assess strength of recommendation [21].

### Data collection

**Figure 1:**
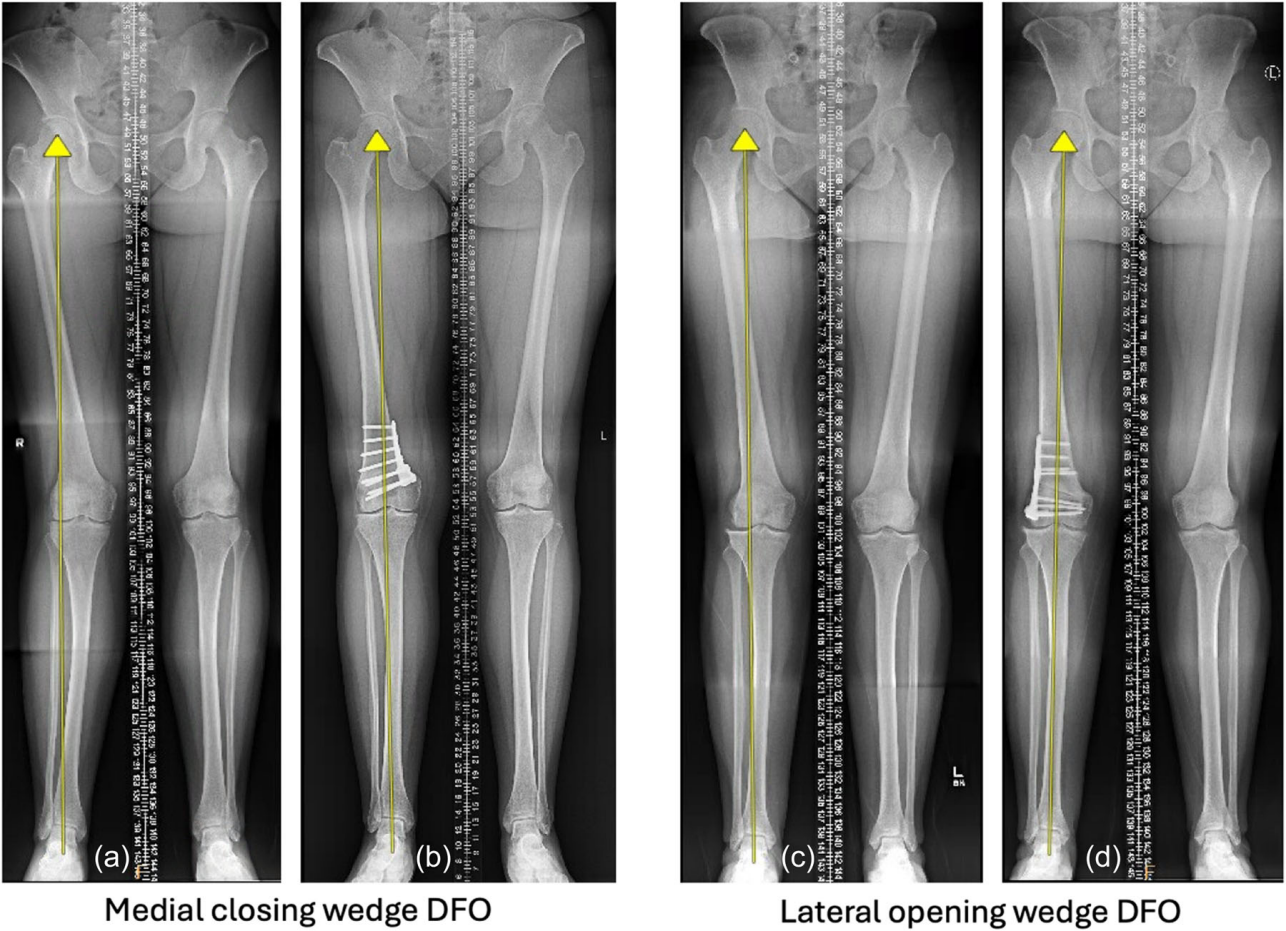
Radiographic examples of pre‐ and post‐operative distal femoral osteotomy (DFO) for correction of valgus alignment with lateral compartment disease. Panels (a) and (b) demonstrate pre‐ and post‐operative medial closing wedge DFO, respectively. Panels (c) and (d) demonstrate pre‐ and post‐operative lateral opening wedge DFO, respectively. Arrows drawn in each radiograph depict the mechanical weightbearing (Mikulicz) line.

Data were collected in Excel (Microsoft Corp) and consisted of author names, year of publication, journal, number of patients on which follow‐up was obtained, age at time of procedure, follow‐up length, gender, type of osteotomy (e.g., medial closing wedge [MCW] or lateral opening wedge [LOW]) (Figure 1), concomitant procedures and postoperative weightbearing protocol. Early weightbearing was classified as any immediate permissible weightbearing postoperatively (WBAT or PWB [e.g., a defined weight in pounds or kilograms, or percentage of bodyweight]). Delayed weightbearing was classified as an initial post‐operative period of either NWB or TTWB. Outcomes consisted of incidence of nonunion, delayed union, loss of fixation or deformity correction, knee stiffness, VTE (both deep vein thrombosis and pulmonary embolism) and PROMs. Collected PROMs included visual analogue scale (VAS) for pain, Knee Injury and Osteoarthritis Outcome Score (KOOS), International Knee Documentation Committee (IKDC) score, Lysholm score, Tegner activity scale, as well as any other validated knee‐related or health‐related PROM. Median and ranges were converted to mean and standard deviation using previously validated methods [24, 45].

### Statistical analysis

Baseline characteristics were reported as weighted means. No comparative meta‐analysis could be performed due to the lack of comparative studies, and therefore a meta‐analysis was performed with a random effects model using R software with meta packages (version 4.2.3; R Foundation for Statistical Computing). Heterogeneity was determined using *I* ^2^ values, and heterogeneity less than 25% was considered low [6]. Forest plots were performed for individual outcomes per treatment group using a random effects model with 95% confidence intervals (CIs). Statistical comparison was limited by low event frequency, and thus no statistically significant associations were identified, and *p*‐values were omitted.

## RESULTS

### Study selection

**Figure 2:**
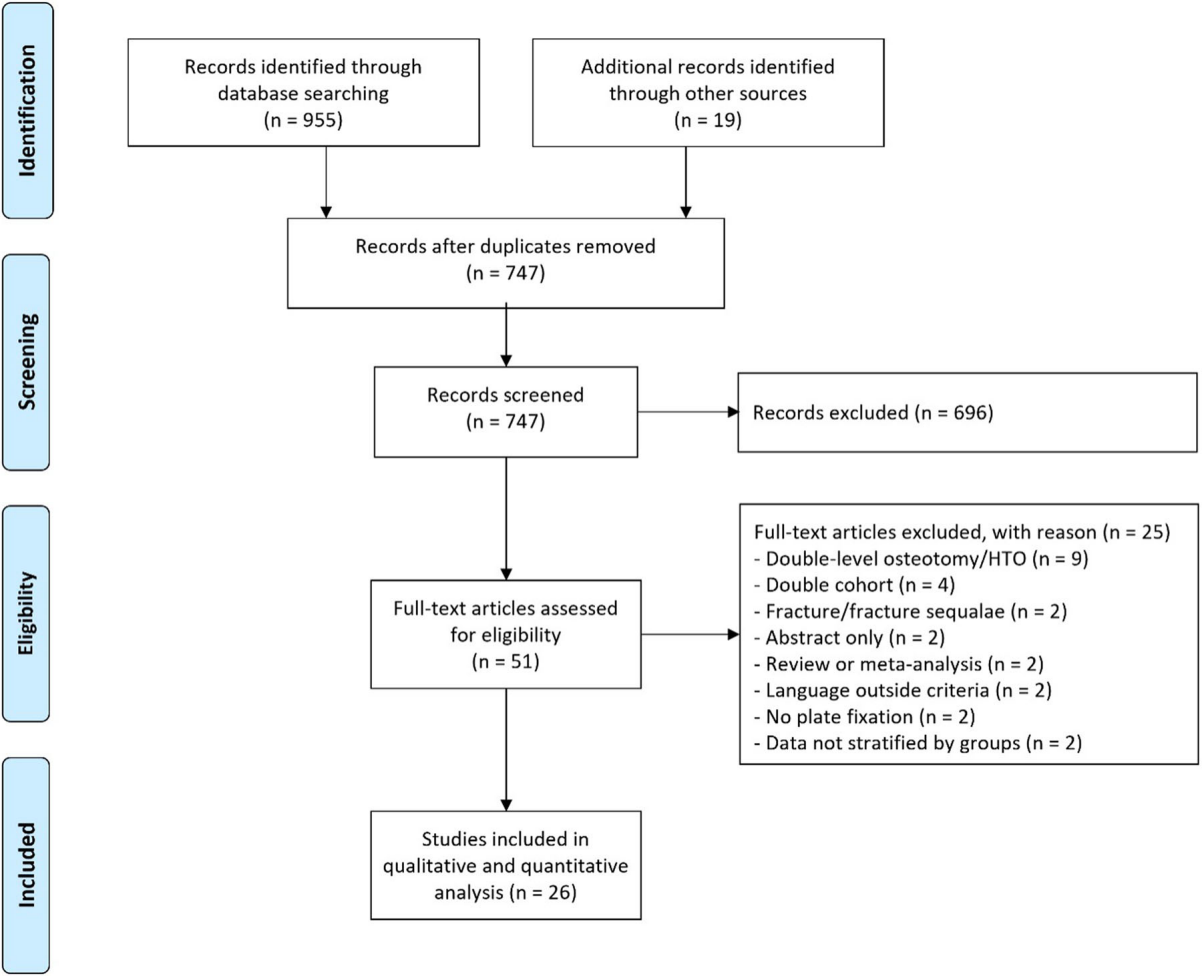
Preferred Reporting Items for Systematic Reviews and Meta‐Analyses (PRISMA) flow diagram of the systematic review. HTO, high tibial osteotomy.

After duplicate removal, 747 studies were reviewed for the first screening and 51 studies reviewed for their full text. A total of 26 studies were included [2, 7, 8, 9, 10, 12, 14, 15, 17, 18, 20, 22, 25, 26, 29, 31, 32, 33, 34, 36, 38, 39, 43, 44, 46, 49], of which 14 studies reporting on medial closing wedge DFO (MCW‐DFO) [2, 17, 18, 20, 22, 25, 26, 31, 32, 33, 34, 36, 43, 46] and 12 studies on lateral opening wedge DFO (LOW‐DFO) [7, 8, 9, 10, 12, 14, 15, 29, 38, 39, 44, 49] (Figure 2). Demographic data are presented in Table 1.

**Table 1:** Baseline characteristics of the included studies.

Authors	Year	No. knees	Age (years)		Follow‐up (years)		Female gender (%)	Osteotomy type	Plate fixation
			Mean	Range	Mean	Range			
MCW—Early weightbearing									
Huang et al. [25]	2021	25	64	47–80	1.4	1.0–3.1	87	MCW	Locking plate (PSI)
Maione et al. [32]	2024	34	49	45–55	9.4	4.4–12.0	27	MCW	Locking plate (Tomofix)
Forkel et al.[18]	2015	22	47	25–55	3.5	‐	74	MCW	Locking plate (Tomofix)
Ismailidis et al. [26]	2023	28	44	17–63	1.8	0.8–6.8	77	22 MCW, 6 LOW	Locking plate (Tomofix)
Lobenhoffer et al. [31]	2017	60	40	17–79	1.8	0.3–3.8	42	MCW	Locking plate (Tomofix)
Stähelin and Hardegger [42]	2004	34	52	‐	4.4	1–12	‐	MCW	Tubular plate
Nha et al. [34]	2021	55	38	16–69	2.9	1.1–7.3	49	MCW	Various plates
MCW—Delayed weightbearing									
Nakamura et al. [33]	2017	5	66	58–74	5.5	5.0–6.2	80	MCW	Locking plate (NCB‐PT)
Backstein et al. [2]	2007	40	44	20–67	10.3	3.3–30.4	74	MCW	90° blade plate
Finkelstein et al. [17]	1996	21	56	27–77	11.1	8.1–20	70	MCW	90° blade plate
Gupta et al. [20]	2014	46	17	15–23	1.7	1.3–2.4	83	MCW	Non‐locking plate
Healy et al. [22]	1988	23	50	19–70	4.0	2.0–9.0	78	20 MCW, 3 LOW	90° blade plate
Omidi‐Kashani et al. [36]	2009	23	23	17–41	1.4	0.8–2.1	78	MCW	90° blade plate
Wang and Hsu [46]	2005	30	53	31–64	8.3	5.1–14.1	93	MCW	90° blade plate
LOW—Early weightbearing									
Elattar et al. [15]	2017	41	44	22–72	2.2	1.0–4.8	79	LOW	Locking plate (Tomofix)
Ekeland et al. [14]	2016	24	48	31–62	7.9	4.0–10.2	46	LOW	Puddu
Liska et al. [29]	2018	98	32	‐	‐	>1.0	58	LOW	Locking plate (Tomofix)
Das [10]	2008	12	55	46–71	2.8	0.9–4.1	58	LOW	Puddu
LOW—Delayed weightbearing									
Dewilde et al. [12]	2013	16	47	30–51	5.7	2.6–10.6	68	LOW	Puddu
Saithna et al. [39]	2013	21	41	28–58	4.5	1.6–9.2	43	LOW	Tomofix & Puddu
Cameron et al. [7]	2015	31	35	‐	5.0	2–12	65	LOW	Various plates
Puzzitiello et al. [38]	2020	32	31	17–47	7.1	2.2–13.3	66	LOW	Unspecified
de Carvalho et al. [9]	2014	26	49	21–65	4.0	1.7–9.5	69	LOW	DCS plate
Cance et al. [8]	2024	38	48	23–61	15.2	10–29	63	LOW	95° blade plate
Thein et al. [44]	2012	7	47	‐	6.5	‐	83	LOW	Puddu plate
Zarrouk et al. [49]	2010	22	53	27–66	‐	>3.0	65	LOW	95° blade plate
Total MCW—EWB	‐	258	45.6	38–64	3.5	1.4–9.4	54	‐	‐
Total MCW—DWB	‐	363	39.0	23–66	5.9	1.4–11.1	80	‐	‐
Total LOW—EWB	‐	176	38.5	32–55	4.1	2.2–7.9	60	‐	‐
Total LOW—DWB	‐	193	42.9	31–53	7.6	4.0–15.2	64	‐	‐
Total all studies	‐	814	41.9	23–66	5.2	1.4–15.2	64	‐	‐

Abbreviations: DCS, dynamic condylar screw; DWB, delayed weightbearing; EWB, early weightbearing; LOW, lateral opening wedge; MCW, medial closing wedge; NCB‐PT, non‐contact bridging for the proximal tibia (Zimmer Biomet); No, number; PSI, patient‐specific instrumentation.

### Quality of studies

There were 3 level III [7, 32, 34] studies and 23 level IV [2, 8, 9, 10, 12, 14, 15, 17, 18, 20, 22, 25, 26, 29, 31, 33, 36, 38, 39, 43, 44, 46, 49] studies. For methodologic study quality as measured by the MINORS criteria, 22/23 (96%) of the non‐comparative studies were of moderate quality and 1/23 (4%) was poor quality, while all 3 (100%) of the comparative studies were of moderate quality (Table 2). Thus, 25/26 (96%) of studies had moderate quality methodology by MINORS criteria. All studies were graded low or very low strength of recommendation by GRADE criteria due to the lack of comparative studies.

**Table 2:** Quality assessment of the included studies using the Methodological Index for NonRandomized Studies (MINORS) criteria.

Authors	Year	Journal	LoE	Study design	1	2	3	4	5	6	7	8	9	10	11	12	Total
Huang et al. [25]	2021	*J Pers Med*	IV	Case series	2	2	2	2	1	1	2	0	–	–	–	–	12
Maione et al. [32]	2024	*Am J Sports Med*	III	Cohort	2	2	1	2	0	2	1	0	2	2	1	2	17
Forkel et al. [18]	2015	*KSSTA*	IV	Case series	2	2	1	2	0	2	2	0	–	–	–	–	11
Ismailidis et al. [26]	2023	*AOTS*	IV	Case series	2	2	1	2	0	1	2	0	–	–	–	–	10
Lobenhoffer et al. [31]	2017	*Oper Orthop Traumatol*	IV	Case series	2	0	0	2	0	1	0	0	–	–	–	–	5
Stähelin and Hardegger [42]	2004	*Der Orthopäde*	IV	Case series	2	2	0	2	0	2	1	0	–	–	–	–	9
Nha et al. [34]	2021	*Am J Sports Med*	III	Cohort	2	2	1	2	1	2	1	1	2	2	2	2	20
Nakamura et al. [33]	2017	*Knee Surg Relat Res*	IV	Case series	2	2	2	1	0	2	2	0	–	–	–	–	11
Backstein et al. [2]	2007	*J Arthroplasty*	IV	Case series	2	2	0	2	0	2	1	0	–	–	–	–	9
Finkelstein et al. [17]	1996	*J Bone Jt Surg Am*	IV	Case series	2	1	2	2	0	2	1	0	–	–	–	–	10
Gupta et al. [20]	2014	*Acta Orthop Belg*	IV	Case series	2	2	2	2	0	1	2	0	–	–	–	–	11
Healy et al. [22]	1988	*J Bone Jt Surg Am*	IV	Case series	1	2	0	1	0	2	2	1	–	–	–	–	9
Omidi‐Kashani et al. [36]	2009	*J Orthop Surg Res*	IV	Case series	2	2	2	2	0	1	2	0	–	–	–	–	11
Wang and Hsu [46]	2005	*J Bone Jt Surg Am*	IV	Case series	2	2	2	2	0	2	2	0	–	–	–	–	12
Elattar et al. [15]	2017	*HSS J*	IV	Case series	2	2	0	2	0	2	1	2	–	–	–	–	11
Ekeland et al. [14]	2016	*KSSTA*	IV	Case series	2	2	2	2	0	2	2	0	–	–	–	–	12
Liska et al. [29]	2018	*KSSTA*	IV	Case series	2	2	1	2	0	0	2	0	–	–	–	–	9
Das [10]	2008	*Open Access Surg*	IV	Case series	2	2	0	2	0	2	1	0	–	–	–	–	9
Dewilde et al. [12]	2013	*KSSTA*	IV	Case series	2	2	2	2	0	2	1	0	–	–	–	–	11
Saithna et al. [39]	2013	*The Knee*	IV	Case series	2	2	2	2	0	2	1	0	–	–	–	–	11
Cameron et al. [7]	2015	*Clin Orthop Relat Res*	III	Cohort	2	2	1	2	0	2	2	0	2	2	1	1	17
Puzzitiello et al. [38]	2020	*Orthop J Sports Med*	IV	Case series	2	2	2	2	0	2	2	0	–	–	–	–	12
de Carvalho et al. [9]	2014	*KSSTA*	IV	Case series	2	2	2	2	0	2	1	0	–	–	–	–	11
Cance et al. [8]	2024	*KSSTA*	IV	Case series	2	2	0	2	0	2	2	0	–	–	–	–	10
Thein et al. [44]	2012	*J Orthop Sci*	IV	Case series	2	2	2	2	1	2	2	0	–	–	–	–	13
Zarrouk et al.[49]	2010	*OTSR*	IV	Case series	2	2	2	2	0	2	2	0	–	–	–	–	12

*Note*: The criteria of MINORS [41] with 0 points when not reported, 1 when reported but inadequate, and 2 when reported and adequate; maximum score, 16 for non‐comparative studies and 24 for comparative studies. (1) A clearly stated aim: the question addressed should be precise and relevant in the light of available literature. (2) Inclusion of consecutive patients: all patients potentially fit for inclusion (satisfying the criteria for inclusion) have been included in the study during the study period (no exclusion or details about the reasons for exclusion). (3) Prospective collection of data: data were collected according to a protocol established before the beginning of the study. (4) Endpoints appropriate to the aim of the study: unambiguous explanation of the criteria used to evaluate the main outcome, which should be in accordance with the question addressed by the study. In addition, the endpoints should be assessed on an intention‐to‐treat basis. (5) Unbiased assessment of the study endpoint: blind evaluation of objective endpoints and double‐blind evaluation of subjective endpoints. Otherwise, the reasons for not blinding should be stated. (6) Follow‐up period appropriate to the aim of the study: the follow‐up should be sufficiently long to allow the assessment of the main endpoint and possible adverse events. (7) Loss to follow‐up: all patients should be included in the follow‐up. Otherwise, the proportion lost to follow‐up should not exceed the proportion experiencing the major endpoint. (8) Prospective calculation of the study size: information of the size of detectable difference of interest with a calculation of 95% CI, according to the expected incidence of the outcome event, and information about the level for statistical significance and estimates of power when comparing the outcomes. (9) An adequate control group: having a gold standard diagnostic test or therapeutic intervention recognised as the optimal intervention according to the available published data. (10) Contemporary groups: control and studies group should be managed during the same period. (11) Baseline equivalence of groups: the groups should be similar regarding the criteria other than the studied endpoint. Absence of confounding factors that could bias the interpretation of the results. (12) Adequate statistical analyses whether statistics were in accordance with type of study with calculation of confidence intervals or relative risk. Abbreviations: *Am J Sports Med, American Journal of Sports Medicine; AOTS, Archives of Orthopaedic and Trauma Surgery; Clin Orthop Relat Res, Clinical Orthopaedics and Related Research; HSS J, The Musculoskeletal Journal of Hospital for Special Surgery; J Arthroplasty, The Journal of Arthroplasty; J Bone Jt Surg Am, The Journal of Bone and Joint Surgery, American Volume; J Orthop Sci, Journal of Orthopaedic Science; J Orthop Surg Res, Journal of Orthopaedic Surgery and Research; J Pers Med, Journal of Personalised Medicine; Knee Surg Relat Res, Knee Surgery & Related Research; KSSTA, Knee Surgery, Sports Traumatology, Arthroscopy;* LoE, level of evidence; *Open Access Surg, Open Access Surgery; Oper Orthop Traumatol, Operative Orthopaedics and Traumatology; Orthop J Sports Med, Orthopaedic Journal of Sports Medicine; OTSR, Orthopaedics & Traumatology: Surgery & Research;* RCT, randomised controlled trial.

### Baseline characteristics

The 26 studies included 814 patients with a mean age of 42 years (range 23–66 years) and of which 64% were female. Mean follow‐up time was 5.2 years (range 1.4–15.2 years). The TomoFix® locking plate (DePuy Synthes, Johnson & Johnson MedTech) was the most frequently used implant, followed by a 90° or 95° blade plate, and the Puddu plate® (Arthrex, Inc.). Regarding postoperative weightbearing, studies were classified into four groups: (I) MCW‐DFO with early weightbearing; (II) MCW‐DFO with delayed weightbearing; (III) LOW‐DFO with early weightbearing; and (IV) LOW‐DFO with delayed weightbearing. A graphical representation of postoperative weightbearing regimens from different studies after MCW‐DFO and LOW‐DFO is included in Supporting Information S1: Figures S1 and S2, respectively. PWB was specified in seven studies and ranged from 10 to 20–kg of bodyweight (Table 1).

### Nonunion, delayed union and loss of fixation or deformity correction

**Figure 3:**
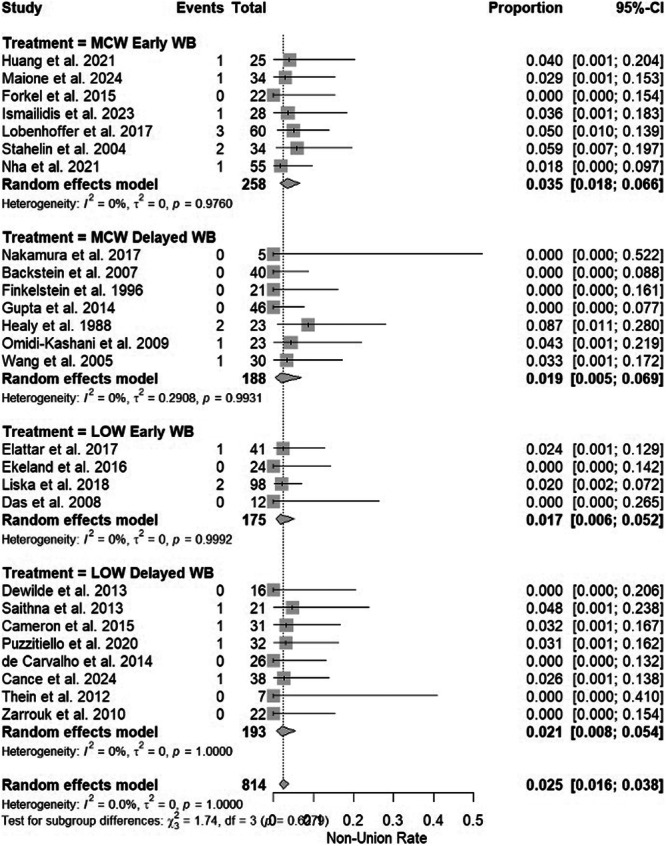
Forest plots of the different studies and treatment groups for incidence of the primary outcome of nonunion. CI, confidence interval; LOW, lateral opening wedge; MCW, medial closing wedge; WB, weightbearing.

Overall, the cumulative incidence of DFO nonunion was 2.5% (95% CI 1.6%–3.8%). Nonunion rates were as follows: MCW‐DFO early weightbearing (3.5%, 95% CI 1.8%–6.6%), MCW‐DFO delayed weightbearing (1.9%, 95% CI 0.5%–6.9%), LOW‐DFO early weightbearing (1.7%, 95% CI 0.6%–5.2%) and LOW‐DFO delayed weightbearing (2.1%, 95% CI 0.8%–5.4%) (Figure 3). An overall low incidence of delayed union was noted at 0.6% (95% CI 0.1%–3.3%). Delayed union rates were as follows: MCW‐DFO early weightbearing (0.5%, 95% CI 0%–16.4%), MCW‐DFO delayed weightbearing (0%, 95% CI 0%–83.9%), LOW‐DFO early weightbearing (5.1%, 95% CI 2.7%–9.6%) and LOW‐DFO delayed weightbearing (0.5%, 95% CI 0.1%–3.6%). Overall incidence of loss of fixation or deformity correction was 1.4% (95% CI 0.5%–3.5%). Loss of fixation or deformity correction rates were as follows: MCW‐DFO early weightbearing (2.2%, 95% CI 0.6%–7.3%), MCW‐DFO delayed weightbearing (1.1%, 95% CI 0.3%–4.2%), LOW‐DFO early weightbearing (0.2%, 95% CI 0%–45%) and LOW‐DFO delayed weightbearing (2.3%, 95% CI 0.5%–9.4%).

### Stiffness and venous thromboembolism

Knee stiffness was reported in 21 studies (579 patients) with an overall incidence of 2.9% (95% CI 1.4%–6.1%). The rates of knee stiffness were as follows: MCW‐DFO early weightbearing (1.0%, 95% CI 0.2%–3.9%), MCW‐DFO delayed weightbearing (4.0%, 95% CI 1.1%–13.7%), LOW‐DFO early weightbearing (2.6%, 95% CI 0.7%–9.8%), LOW‐DFO delayed weightbearing (8.3%, 95% CI 4.1%–16.4%). VTE was reported in 14 studies (462 patients) with an overall incidence of 0.9% (95% CI 0.3%–2.3%).

### Patient‐reported outcome measures

Eleven out of 26 studies (42%) reported validated PROMs, but the specific instruments used varied substantially across studies, including KOOS (four studies), IKDC (three studies), Lysholm (three studies), Tegner activity scale (three studies), Oxford Knee Score (three studies), 36‐item short form health survey (SF‐36) (two studies) and VAS for pain (one study). The heterogeneity in PROM instruments, scoring scales and follow‐up time points precluded meaningful quantitative pooling of data. Consequently, a meta‐analysis was not performed for PROMs.

## DISCUSSION

The most important finding of the present systematic review was that DFO demonstrated low rates of major mechanical and medical complications across different post‐operative weightbearing protocols. The cumulative incidence of the primary outcome of nonunion was 2.5%, with low rates observed across MCW‐DFO and LOW‐DFO regardless of early or delayed weightbearing. Delayed union was uncommon (0.6% overall), and loss of fixation or deformity correction occurred infrequently (1.4%), suggesting that contemporary fixation strategies provide reliable mechanical stability even with early mobilisation. Postoperative knee stiffness was also relatively rare (2.9% overall), with highest rates reported after LOW‐DFO with delayed weightbearing. The incidence of VTE was low (0.9%), supporting the overall safety of DFO when modern perioperative VTE protocols are employed. Collectively, these findings indicate that DFO is associated with a favourable complication profile, with no clear sign of increased risk attributable to early weightbearing.

A prior systematic review in 2016 found overall rates of delayed union, nonunion and loss of correction after DFO to be 3.8%, 3.2% and 3.0% respectively, which are slightly higher than the rates identified in the present systematic review [48]. The discrepancy in complication rates may be due to inclusion of newer studies with lower complication rates relating to union and deformity correction [8, 25, 31, 32, 33, 34, 38].

Early weightbearing after DFO has theoretical risks of delayed union, nonunion and loss of deformity correction or fixation. The rates of delayed union were 0.5%–5.1% after early weightbearing and 0%–0.5% after delayed weightbearing. A prior systematic review found a delayed union rate of 5.8% after LOW‐DFO and 3.8% after MCW‐DFO [48]. Another systematic review found a delayed union rate of 2% after LOW‐DFO and 0% after MCW‐DFO [13]. Neither of these two prior systematic reviews stratified delayed union rates based on postoperative weightbearing. The rare events of nonunion and delayed union after DFO are also influenced by factors other than weightbearing (e.g., presence of hinge fracture [40], opening wedge gap size [19], smoking [23, 29]), so any association between weightbearing and union rate in these retrospective data should be interpreted with caution. In the field of orthopaedic trauma, a retrospective cohort study found no differences in rates of nonunion or malunion with early versus delayed weightbearing after locked lateral plating of distal femur fractures [43]. Older patients with traumatic distal femur fractures represent a different patient population than those undergoing DFO for localised osteoarthritis or deformity correction, but these data add to the growing body of evidence of the safety of early weightbearing with locked lateral plating of the distal femur.

The overall mean rate of knee stiffness in the present review was 2.9% after DFO, with a range of 0%–20%. There is a widely reported range of postoperative knee stiffness rates after DFO in the literature. A single‐institution case series of 69 patients undergoing DFO identified a knee stiffness rate of 29%, which is tenfold higher than the mean rate identified in the present systematic review [4]. The patients in this case series underwent a delayed weightbearing protocol: 4–6 weeks nonweightbearing, followed by PWB, and incremental advancement towards WBAT by 12 weeks. The case series was excluded from the present systematic review due to a lack of minimum 1 year follow‐up, but if included would have increased the overall reported mean knee stiffness rate.

The overall rate of VTE after DFO in the present review was relatively low (0.9%) and comparable to two prior systematic reviews comparing MCW and LOW DFO which reported VTE rates of 0.5%–1% [13, 48]. There was no standardised VTE prophylaxis given across studies identified in this review, and there is currently no consensus or established guidelines on postoperative VTE prophylaxis after DFO. While the rates of symptomatic DVT after osteotomies around the knee are relatively low, a prospective cohort study found an asymptomatic distal (calf vein) DVT rate of 21 out of 93 (22.6%) at 1 week postoperatively after lateral closing wedge DFO [37]. These data suggest that there is a potentially higher rate of unreported asymptomatic VTE among patients undergoing osteotomies around the knee than is currently recognised.

The highest reported rate of postoperative knee stiffness from a single study identified in the present review was 17% in a retrospective case series of 30 patients undergoing MCW‐DFO [46]. MCW‐DFO may also have a theoretically higher risk of postoperative AMI compared to LOW‐DFO due to elevation and retraction of the VMO during the MCW‐DFO surgical approach, which could contribute to knee stiffness.

### Limitations

The most notable limitation of this systematic review is a lack of high‐level evidence in the identified literature, with 23 level IV studies and 3 level III studies, and 96% of studies having only moderate quality methodology by MINORS criteria. The overall low level of evidence makes it impossible to draw definitive conclusions between the effects of early versus delayed weightbearing after DFO, as these studies are not directly comparing two variables and controlling for confounders. Any observed differences between early and delayed weightbearing in this systematic review may therefore be artifactual or reflect surgeon preference or selection bias, rather than true biologic effects. Due to the lack of comparative studies identified, a meta‐analysis with a random effects model was utilised in this systematic review. Another major limitation is the lack of PROM data in the identified studies, making it impossible to answer the secondary outcome question regarding the association between early versus delayed weightbearing and PROMs, which is arguably the most clinically meaningful outcome. Figure 3 reports pooled estimates from the meta‐analysis with random effects models, but the lack of prospective comparative evaluations between early and delayed weightbearing within matched populations limit the conclusions which can be drawn from a pooled estimate. Lastly, the inclusion of multiple studies >20 years old [26, 46] may limit comparisons to modern‐day surgical techniques, though each of these studies still utilised locking plate technology for internal fixation of the osteotomy.

## CONCLUSIONS

There were relatively low overall mean rates of delayed union, nonunion, loss of fixation or deformity correction, and VTE after DFO, regardless of an early or delayed post‐operative weightbearing protocol. Due to limited comparative data and the risk of selection bias, definitive conclusions cannot be drawn regarding the safety of early weightbearing after DFO, underscoring the need for prospective controlled studies.

## AUTHOR CONTRIBUTIONS

Concept and design done by Nicholas A. Apseloff, Jelle P. van der List, Christopher C. Kaeding, Robert A. Magnussen and David C. Flanigan. Literature search done by Nicholas A. Apseloff, Jelle P. van der List and Tarik Taoufik. Study selection done by Nicholas A. Apseloff, Jelle P. van der List and Tarik Taoufik. Data extraction done by Jelle P. van der List and Tarik Taoufik. Quality/risk‐of‐bias assessment done by Nicholas A. Apseloff and Tarik Taoufik. Statistical analysis and data synthesis done by Nicholas A. Apseloff and Jelle P. van der List. Manuscript drafting: Nicholas A. Apseloff and Jelle P. van der List. Critical revisions of the manuscript for important intellectual content: Nicholas A. Apseloff, Jelle P. van der List, Christopher C. Kaeding, Robert A. Magnussen and David C. Flanigan. Final approval of the manuscript done by all authors.

## CONFLICT OF INTEREST STATEMENT

The authors declare no conflicts of interest.

## ETHICS STATEMENT

The authors have nothing to report.

## Supporting information


**Supplement 1.** An overview of the different postoperative regimens for all medial closing wedge distal femoral osteotomy studies.
**Supplement 2.** An overview of the different postoperative regimens for all lateral opening wedge distal femoral osteotomy studies.

## Data Availability

The data that support the findings of this study are available from the corresponding author upon reasonable request.
