# Supplementary material for: Postoperative weightbearing timing after distal femur osteotomy is not associated with rates of union or complications: A systematic review and meta‐analysis
Source: Knee Surg Sports Traumatol Arthrosc. 2026 Feb 16;34(5):1776–86. doi: 10.1002/ksa.70340 (PMC13122738; doi:10.1002/ksa.70340)
Supplement: Supplementary file 1 — Supplement 1. An overview of the different postoperative regimens for all medial closing wedge distal femoral osteotomy studies. Supplement 2. An overview of the different postoperative regimens for all lateral opening wedge distal femoral osteotomy studies. [file KSA-34-1776-s001.docx]

**
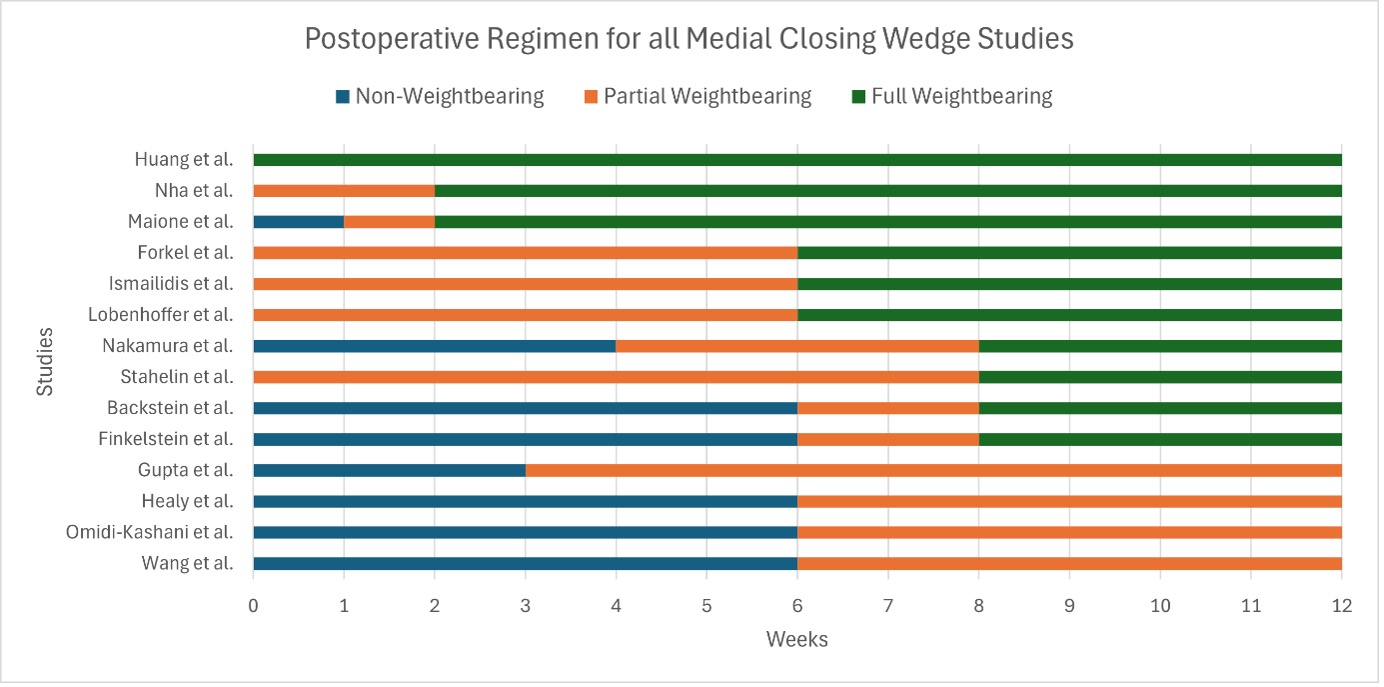
Supplement 1.** An overview of the different postoperative regimens for all medial closing wedge distal femoral osteotomy studies.

**
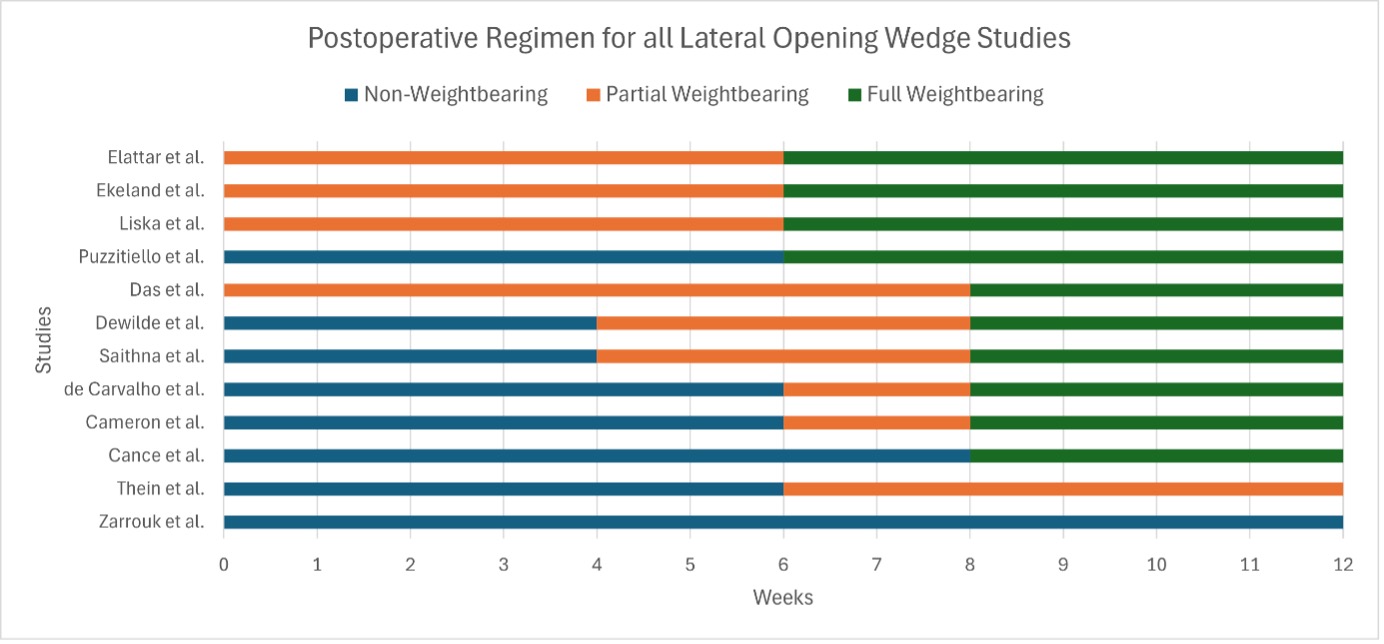
**

**Supplement 2.** An overview of the different postoperative regimens for all lateral opening wedge distal femoral osteotomy studies.
